# The Role of Sphingosine Kinase 1/Sphingosine-1-Phosphate Pathway in the Myogenic Tone of Posterior Cerebral Arteries

**DOI:** 10.1371/journal.pone.0035177

**Published:** 2012-04-20

**Authors:** Mihwa Lim, Soo-Kyoung Choi, Young-Eun Cho, Soo-In Yeon, Eok-Cheon Kim, Duck-Sun Ahn, Young-Ho Lee

**Affiliations:** Department of Physiology, College of Medicine, BK 21 Project for Medical Sciences, Yonsei University, Seoul, Korea; Osaka University Graduate School of Medicine, Japan

## Abstract

**Aims:**

The goal of the current study was to determine whether the sphingosine kinase 1 (SK1)/sphingosine-1-phosphate (S1P) pathway is involved in myogenic vasoconstriction under normal physiological conditions. In the present study, we assessed whether endogenous S1P generated by pressure participates in myogenic vasoconstriction and which signaling pathways are involved in SK1/S1P-induced myogenic response under normal physiological conditions.

**Methods and Results:**

We measured pressure-induced myogenic response, Ca^2+^ concentration, and 20 kDa myosin light chain phosphorylation (MLC_20_) in rabbit posterior cerebral arteries (PCAs). SK1 was expressed and activated by elevated transmural pressure in rabbit PCAs. Translocation of SK1 by pressure elevation was blocked in the absence of external Ca^2+^ and in the presence of mechanosensitive ion channel and voltage-sensitive Ca^2+^ channel blockers. Pressure-induced myogenic tone was inhibited in rabbit PCAs treated with sphingosine kinase inhibitor (SKI), but was augmented by treatment with NaF, which is an inhibitor of sphingosine-1-phosphate phosphohydrolase. Exogenous S1P further augmented pressure-induced myogenic responses. Pressure induced an increase in Ca^2+^ concentration leading to the development of myogenic tone, which was inhibited by SKI. Exogenous S1P further increased the pressure-induced increased Ca^2+^ concentration and myogenic tone, but SKI had no effect. Pressure- and exogenous S1P-induced myogenic tone was inhibited by pre-treatment with the Rho kinase inhibitor and NADPH oxidase inhibitors. Pressure- and exogenous S1P-induced myogenic tone were inhibited by pre-treatment with S1P receptor blockers, W146 (S1P1), JTE013 (S1P2), and CAY10444 (S1P3). MLC_20_ phosphorylation was increased when the transmural pressure was raised from 40 to 80 mmHg and exogenous S1P further increased MLC_20_ phosphorylation. The pressure-induced increase of MLC_20_ phosphorylation was inhibited by pre-treatment of arteries with SKI.

**Conclusions:**

Our results suggest that the SK1/S1P pathway may play an important role in pressure-induced myogenic responses in rabbit PCAs under normal physiological conditions.

## Introduction

The myogenic response is an intrinsic vascular response characterized by vasoconstriction in response to an increase in intravascular pressure and vasodilation in response to a decrease in intravascular pressure [1]. Arterial myogenic tone plays an important role in establishing ambient vascular tone and auto-regulating blood flow in the resistance vasculature, especially in cerebral circulation [2]–[4], because cerebral arteries are not particularly responsive to the sympathetic nerves surrounding them [5].

The biologically active sphingomyelin metabolite, sphingosine-1-phosphate (S1P), generated by the enzyme sphingosine kinase 1 (SK1), is present in plasma at high nanomolar concentrations, released from activated platelets [6], [7], and found in increased quantities in inflammation and atherosclerosis [8]. S1P plays an important role as a vascular modulator [9]–[11], and most effects of S1P are mediated by a family of five highly specific G-protein–coupled receptors called S1P receptors [12]. It was reported that the myogenic responses of isolated resistance arteries were increased in the smooth muscle cells of SK1-transfected arteries [12], [13]. It was also reported that myogenic vasoconstriction in response to increased transmural pressure was significantly reduced in resistance arteries transfected with sphingosine-1-phosphate phosphohydrolase 1 (SPP1), a S1P-degrading enzyme [14]. Taken together, these results suggest that SK1 and its product, S1P, may be involved in the pressure-induced signaling cascade leading to myogenic vasoconstriction. However, whether SK1/S1P contributes to pressure-induced myogenic responses under normal physiological conditions is unknown.

Cerebral vasospasm is a sustained abnormal contraction of the cerebral arteries [15], [16]. Several spasmogenic substances including oxyhemoglobin, endothelin-1, thrombin, serotonin, noradrenalin, and thromboxane have been suggested [17], [18]. Sphingolipids have been suggested as candidate spasmogenic substances because they are released from activated platelets and are found at high levels in inflammation and predisposing situations for vasospasm [17]. Therefore, the changes in vascular contractility, including myogenic tone, induced by sphingolipids in cerebral arteries should be determined. We already reported the augmentation of cerebral arterial tone by sphingolipid metabolites as well as the underlying mechanisms [19]. Therefore, the role of the SK1/S1P pathway in pressure-induced myogenic tone and the underlying mechanisms remain to be elucidated.

The goal of this study was to determine whether SK1/S1P participates in pressure-induced myogenic responses under normal physiological conditions and which signaling pathways are involved in SK1/S1P-induced myogenic responses. We examined the effect of endogenous and exogenous S1P on myogenic responses. We measured the contribution of endogenous S1P on myogenic response upon vasoconstriction induced by activated SK1 when transmural pressure was increased, and the effect of exogenous S1P was evaluated as vasoconstriction was induced by treatment with S1P in a bath solution at elevated transmural pressure.

## Methods

This investigation conformed to the *Guide for the Care and Use of Laboratory Animals* published by the US National Institutes of Health (NIH publication No. 85–23, revised in 1996). The experimental protocols used in this study were reviewed and approved by the Ethics Committee and Institutional Animal Care and Use Committee of Yonsei University College of Medicine.

### 1.1. Tissue Preparation

New Zealand white rabbits were used in this study and anesthetized with sodium pentobarbital (50 mg/kg, IV injection) containing, heparin (2000 IU/kg), an anticoagulant. The rabbit brains were removed and placed in a normal Krebs-Henseleit (KH) solution composed of the following substances (in mmol/L): NaCl, 119; CaCl_2_, 2.5; NaHCO_3_, 25; MgSO_4_, 1.2; KH_2_PO_4_, 1.2; KCl, 4.6; and glucose, 11.1. The KH solution was continuously aerated with a 95% O_2_-5% CO_2_ gas mixture. The rabbit posterior cerebral arteries (PCAs) were dissected and their segments of about 3–4 mm in length were prepared.

### 1.2. Measurement of Myogenic Tone Using an Arteriograph System

Rabbit PCA segments (100–250 µm inner diameter and 3–4 mm in length) were dissected, cannulated in a pressure myograph (Living Systems Instrumentation, Burlington, VT, USA) filled with KH solution, and subsequently placed on the stage of an inverted microscope (Eclipse TS100/TS100-F, Nikon Inc., Melville, NY, USA). The proximal cannula was connected to a solid-state pressure transducer and reservoir of KH solution, and the transmural pressure was controlled by a pressure servo-controller. The distal cannula was connected to a luer-lock valve that was opened to flush the lumen during the initial cannulation. After cannulation, the valve was closed, and all measurements were conducted under no-flow conditions. The arterial lumen diameter was recorded using the SoftEdge Acquisition Subsystem (IonOptix, Milton, MA, USA).

The KH perfusing and superfusing in the arterial segments was equilibrated with a 95% O_2_-5% CO_2_ gas mixture at 37°C. To eliminate the potential influence of endothelial factors on the pressure-induced myogenic tone, an air bolus was passed through the lumen to disrupt the endothelium. The function of the endothelium was checked at the beginning of each experiment with 10^–6^ M acetylcholine in arteries pre-contracted with 70 mM K^+^ solution (equimolar substitution of Na^+^ with K^+^).

After being mounted, the de-endothelialized cerebral arterial segments were stretched longitudinally to approximate the *in situ* length and were maintained at a transmural pressure of 40 mmHg for a 40–60 min equilibration period. After the equilibration period, the pressure was increased in a stepwise manner from 20 to 100 mmHg in 20-mmHg increments, and each pressure was maintained for 10 min to allow blood vessel diameter to stabilize before measurement. In some experiments, an abbreviated myogenic protocol was used. The arteries were equilibrated and constricted as described above, but the initial pressure was 40 mmHg and was increased to 80 mmHg. After a series of step changes, the transmural pressure was returned to 40 mmHg and the vessel was allowed to re-equilibrate for a minimum of 40 min. At the end of each experiment, a passive pressure-diameter relationship was established in Ca^2+^-free KH solution containing 0.1 µmol/L nifedipine to determine the maximum passive diameter. When the effects of drugs on myogenic responses were assessed, they were administered at a transmural pressure of 40 mmHg 15–30 min before increasing the luminal pressure.

Responses to changes in transmural pressure were normalized as a percentage of the initial diameter at 40 mmHg to control for changes in the resting tone caused by the drugs. The following formula was used to calculate the percent myogenic tone at each pressure step: percent myogenic tone  =  {(*DpX*/*Dp40*) − (*DaX/Da40*)}×100, where *DpX and Dp40* are the passive diameters at a given pressure and 40 mmHg in Ca^2+^-free PSS (0 mM Ca^2+^ with 0.1 µmol/L nifedipine), respectively, and *DaX and Da40* are the active diameters at a given pressure and 40 mmHg in normal PSS in the presence of extracellular Ca^2+^, respectively.

### 1.3. Measurement of Smooth Muscle Ca^2+^ in Pressurized Arteries

Rabbit PCA segments were loaded with the Fura-2AM (10 µmol/L; Molecular Probes, Eugene, OR, USA), the Ca^2+^-sensitive fluorescent indicator, and 0.02% cremophor EL (Sigma, St Louis, MO, USA) in KH as previously reported [20]. The arteries were incubated in this solution for 3 h at room temperature in the dark. Fura-2AM-loaded PCA segments were mounted in a pressure myograph and pressurized to 40 mmHg using a pressure-servo controller and then superfused with KH (37°C) that was aerated with 95% O_2_ + 5% CO_2_ to wash out the excess dye and to allow for hydrolysis of the AM groups by intracellular esterases. The transmural pressure of the arteries was then elevated to 80 mmHg. Fura-2-loaded vessels were alternately excited at 340 and 380 nm at a frequency of 1 Hz with an IonOptix Hyperswitch dual excitation light source, and the respective emissions at 510 nm were detected with a photomultiplier tube. Background-subtracted 340/380 emission ratios were calculated using IonOptix Ion Wizard software and recorded continuously throughout the experiment. The fluorescent emission at 510 nm (R340/380) and the changes in arterial diameter, monitored by video microscopy (IonOptix), were recorded simultaneously.

### 1.4. Immunofluorescence of SK1 in PCA

The localization of SK1 in isolated rabbit PCAs was measured using immunostaining as described previously [21]. PCAs were probed with SK1 antibody (1∶200, Millipore, Temecula, CA, USA).

### 1.5. Western Blotting

The expression of SK1 and S1P receptor proteins in rabbit PCAs were measured by western blot as described previously [19]. Membranes were probed with SK1 antibody (1∶200, Millipore), S1P receptors (1∶200, Millipore), and actin antibody (1∶5,000, Abcam, Cambridge, UK) was used as a loading control.

### 1.6. MLC_20_ Phosphorylation Measurements

Phosphorylation of the 20 kDa myosin light chain (MLC_20_) in the rabbit PCA was measured as described previously [19]. Vessels were rapidly removed from the pressure myograph and frozen when myogenic responses were stable after changes in transmural pressure from 40 mmHg to 80 mmHg. For each preparation, vessels from three to four animals were pooled. Membranes were probed with a specific phosphor-MLC_20_ monoclonal antibody (1∶100; Cell Signaling, Boston, MA, USA) and total MLC_20_ antibody (1∶200; Cell Signaling).

### 1.7. Drugs

The following drugs were used: S1P (Sigma), SKI (EIPA; Sigma, St Louis, MO, USA), NaF (Sigma), (R)-3-Amino-(3-hexylphenylamino)-4-oxobutylphosphonic acid (W146) (Sigma), 1-[1,3-Dimethyl-4-(2-methylethyl)-1*H*-pyrazolo[3,4-*b*]pyridine-6-yl]-4-(3,5-dichloro-4-pyridinyl)-semicarbazide (JTE013) (Tocris Bioscience, Ellisville, MO, USA), 2-undecyl-thiazolidine-4-carboxylic acid (CAY10444) (Cayman Chemical, Ann Arbor, MI, USA), fasudil hydrochloride (Tocris), DPI (Sigma), apocynin (Sigma), 1-[β-[3-(4-methoxyphenyl)propoxy]-4-methoxyphenethyl]-1H-imidazole hydrochloride (SKF-96365) (Tocris Bioscience), 9-phenanthrol (Sigma), and nifedipine (Sigma). The general laboratory reagents used were analytical grade or better. S1P was dissolved in 100% ethanol. SKF96365 and 9-phenanthrol were dissolved in dimethyl sulfoxide (DMSO) to a maximum final DMSO concentration of 0.1% that had no effect on vascular tone (data not shown).

### 1.8. Statistics

All values given in the text were expressed as mean±SEMs and analyzed by one-way and two-way ANOVA, followed by the Student-Newman-Keuls post-hoc test. Differences were considered significant at the *P*<0.05 level.

## Results

### 2.1. Activation of SK1 by Transmural Pressure Elevation

**Figure 1:**
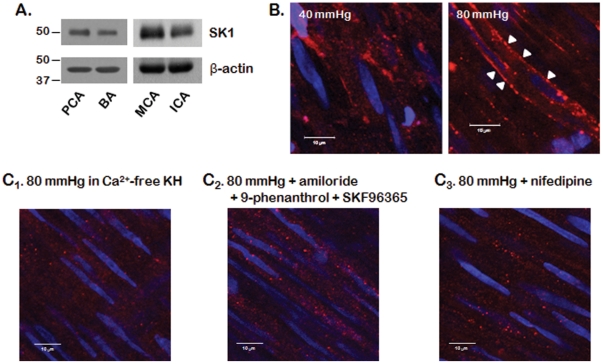
Expression and activation of SK1 by elevated of transmural pressure. A: Western blot analysis of SK1 expression in rabbit vessels. Representative western blots of SK1 and actin in isolated rabbit posterior cerebral arteries (PCAs), basilar arteries (BAs), middle cerebral arteries (MCAs), and internal carotid arteries (ICA). B: SK1 iImmunofluorescence in isolated rabbit posterior cerebral arteries at 40 and 80 mmHg. C: SK1 immunofluorescence at pressure elevation (80 mmHg) in the absence of external Ca^2+^ (C_1_) and in the presence of mechanosensitive ion channel (C_2_), and voltage-sensitive Ca^2+^ channel blockers (C_3_).

We first determined whether SK1 is expressed in rabbit PCAs using western blot analysis. The western blots used to detect SK1 expression in isolated rabbit PCAs are shown in Figure 1A. SK1 antibodies were labeled as a major band around 50 kDa in isolated PCAs, basilar arteries (BAs), middle cerebral arteries (MCAs), and internal carotid arteries (ICAs).

We also determined whether SK1 is activated by pressure elevation. Elevation of transmural pressure from 40 to 80 mmHg was associated with translocation of SK1 from the cytosol to the plasma membrane (Figure 1B). Translocation of SK1 by pressure elevation was blocked in the absence of external Ca^2+^ (Ca^2+^-free KH solution; Figure 1C_1_). Translocation of SK1 by pressure elevation was also blocked in the presence of mechanosensitive ion channel blockers, 1 µmol/L amiloride (epithelial Na^+^ channel blocker), 5 µmol/L 9-phenanthrol (selective TRPM4 blocker), and 10 µmol/L SKF 96365 (nonselective blocker of TRPC channels) (Figure 1C_2_). Nifedipine, a voltage-sensitive Ca^2+^ channel blocker, also blocked translocation of SK1 by elevation of transmural pressure from 40 to 80 mmHg (Figure 1C_3_).

### 2.2. Role of Endogenous and Exogenous S1P in Myogenic Tone

**Figure 2:**
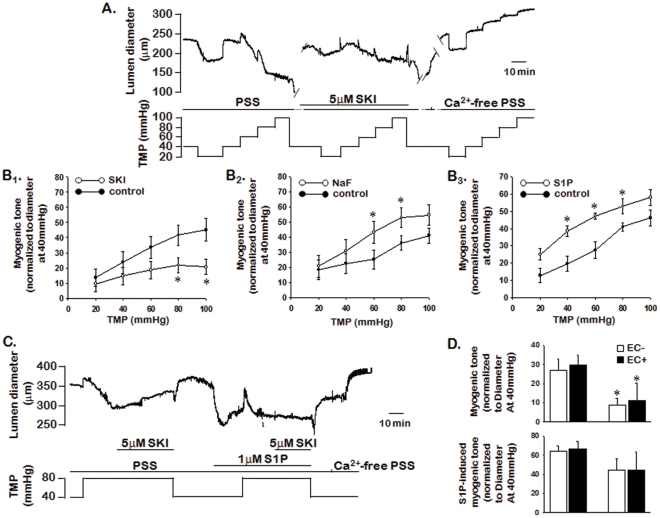
The effect of endogenous and exogenous S1P on myogenic tone. A: Representative traces showing the effect of 5 µmol/L SKI on pressure-induced myogenic tone. B: The mean data for the effects of SKI (B_1_; n = 9), NaF (B_2_; n = 6), and S1P (B_3_; n = 5) on pressure-induced myogenic tone. Changes in the lumen diameter were measured in response to 20 mmHg stepwise increases in transmural pressure in Ca^2+^-containing Kreb’s-Henseleit solution (active tone; KH) or Ca^2+^-free KH solution (passive tone). C: Representative traces from five independent results showing the effect of pre-treatment of S1P on SKI-induced inhibitory effect of myogenic tone. D: Summarized data for changes in myogenic tone during the elevation of transmural pressure (from 40 mmHg to 80 mmHg) and application of S1P in posterior cerebral arteries with and without endothelium. S1P and inhibitors were administered at a transmural pressure of 40 mmHg 30 min before increasing in luminal pressure. Data are expressed as means ± SEMs (n = 5–9) and are normalized to myogenic tone at a pressure of 40 mmHg. *Significantly different compared to the control (*P*<0.05). TMP: transmural pressure. EC: endothelial cells.

To determine whether endogenous S1P generated by transmural pressure elevation is involved in the development of myogenic tone, we evaluated myogenic tone in the presence of 5 µmol/L SKI, an inhibitor of SK1 (Figures 2A & B_1_), and 1 mmol/L NaF, an inhibitor of the S1P-degrading enzyme, sphingosine-1 phosphate phosphohydrolase (SPP) (Figure 2B_2_). The myogenic response induced by increased transmural pressure (stepwise from 20 to 100 mmHg) was decreased in the presence of SKI (n = 9), but it was increased in the presence of NaF (n = 6).

To determine the effect of exogenous S1P on myogenic tone, we evaluated myogenic tone in isolated rabbit PCA in the presence and absence of exogenous S1P (1 µmol/L; Figure 2B_3_). The myogenic response elicited by a pressure increase from 20 to 100 mmHg was elevated in the presence of exogenous S1P (n = 5). We also determined the effect of pre-treatment of S1P on SKI-induced inhibitory effect of myogenic tone. As shown in Figure 2C, the SKI inhibited myogenic responses induced by increased transmural pressure (from 40 to 80 mmHg). Pre-treatment of S1P decreased blood vessel diameter and abolished the SKI-induced inhibitory effect of myogenic tone. These results suggest that an increase in transmural pressure activates SK1, which then increases endogenous S1P. Furthermore, our results suggest that endogenous and exogenous S1P are involved in the development of myogenic tone.

In control experiments, there were no significant differences between two consecutive pressure-induced myogenic responses (data not shown). In addition, to determine the EC_50_ for S1P, SKI, and NaF, we generated a concentration-response curve for these drugs at a 80 mmHg transmural pressure. The EC_50_ values were 1.28 µM for S1P, 5.49 µM for SKI, and 1.01 mM for NaF (data not shown).

To determine the role of endothelium on endogenous and exogenous S1P-induced myogenic tone, we compared myogenic tone between the presence and absence of endothelium in isolated rabbit PCAs. As shown in Figure 2D, raising the transmural pressure from 40 to 80 mmHg developed myogenic tone and pressure-induced myogenic tone was similar between endothelium denuded (27.1 ± 5.8, n = 5) and intact (29.7 ± 5.0, n = 5) arteries. Exogenous S1P-induced myogenic tone was also similar between endothelium denuded (63.9 ± 5.9, n = 5) and intact (66.7 ± 7.5, n = 5) arteries. SKI (1 µmol/L) significantly (*P*<0.05, n = 5) inhibited the pressure-induced development of myogenic tone and the inhibitory effect was not different between endothelium denuded and intact arteries. However, SKI had no significant effect on S1P-induced myogenic tone in endothelium denuded and intact arteries (Figure 2D; n = 5).

### 2.3. Mechanisms Underlying Endogenous and Exogenous S1P-induced Myogenic Tone

**Figure 3:**
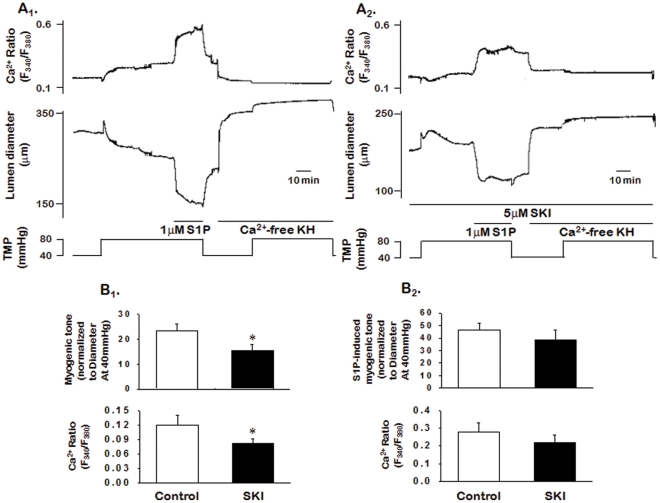
Changes in the Ca^2+^ fluorescence ratio and lumen diameter during transmural pressure elevation and S1P application. A: Representative traces demonstrate changes in the Ca^2+^ ratio and lumen diameter during the elevation of transmural pressure (from 40 to 80 mmHg) and application of S1P in the absence (A_1_) or presence (A_2_) of SKI. B: Summarized data for changes in the Ca^2+^ ratio and myogenic tone during the elevation of transmural pressure (B_1_) and application of S1P (B_2_) in the absence or presence of SKI. Data are expressed as means ± SEMs (n = 6). TMP: transmural pressure.

We first determined the influence of S1P on the changes in Ca^2+^ concentration by measuring vascular wall Ca^2+^ concentration using Fura-2AM, the fluorescence ion indicator. As shown in Figure 3, raising the transmural pressure from 40 to 80 mmHg increased Ca^2+^ fluorescence intensity. Application of exogenous S1P further increased Ca^2+^ fluorescence intensity. The increase in Ca^2+^ fluorescence intensity was accompanied by the development of myogenic tone (Figure 3A_1_; n = 6). To determine whether blockading SK1, the S1P-generating enzyme, with SKI altered the Ca^2+^ fluorescence intensity, we measured the effect of SKI pretreatment on changes in Ca^2+^ fluorescence intensity. SKI (1 µmol/L) significantly (*P*<0.05, n = 6) inhibited the pressure-induced increase in Ca^2+^ fluorescence intensity and development of myogenic tone (Figures 3A_2_ and B_1_). However, SKI had no significant effect on the S1P-induced increase in Ca^2+^ fluorescence intensity and myogenic tone (Figures 3A_2_ and B_2_; n = 5).

**Figure 4:**
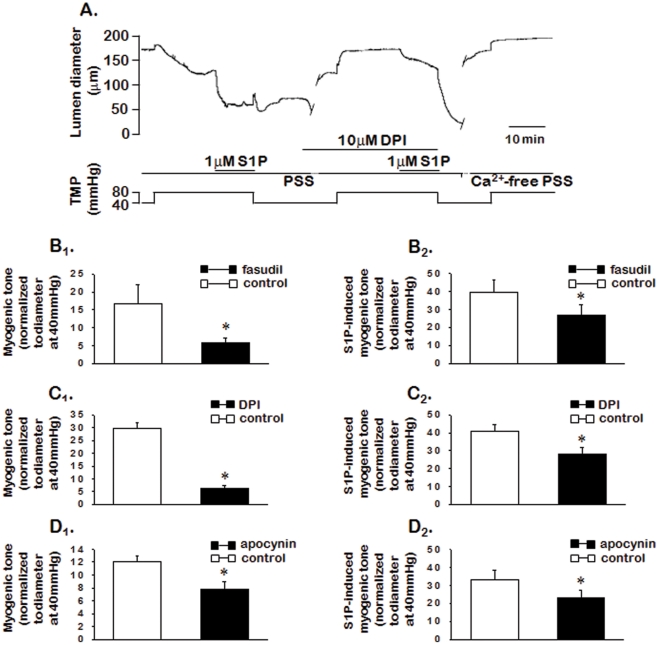
Effects of Rho kinase and NADPH oxidase inhibitors on pressure- and exogenous S1P-induced myogenic tone. A: Representative traces demonstrate changes in lumen diameter during the elevation of transmural pressure (from 40 to 80 mmHg) and application of S1P in the absence or presence of DPI. B: Summarized data for changes in myogenic tone during the elevation of transmural pressure and application of S1P in the absence or presence of fasudil (B; n = 5), DPI (C; n = 6), or apocynin (D; n = 5). Data are expressed as means ± SEMs (n = 5–6). TMP: transmural pressure.

To assess the possible involvement of Ca^2+^ sensitization of the contractile apparatus (Ca^2+^ sensitization) pathway, we evaluated the role of RhoA/Rho kinase and NADPH oxidase/reactive oxygen substrate (ROS) on endogenous and exogenous S1P-induced myogenic tone (Figure 4). Fasudil (1 µmol/L), the Rho kinase inhibitor, significantly (*P*<0.05, n = 5) inhibited pressure- and exogenous S1P-induced myogenic tone (Figures 4B_1_ and B_2_). Inhibition of NADPH oxidase with 10 µmol/L DPI, a broad chemical inhibitor of signaling pathways unrelated to ROS generation, significantly (*P*<0.05, n = 6) reduced pressure- and exogenous S1P-induced myogenic tone (Figures 4A, C_1_ and C_2_). Furthermore, apocynin, a specific chemical inhibitor of NADPH oxidase, also significantly (*P*<0.05, n = 5) reduced pressure- and exogenous S1P-induced myogenic tone (Figures 4D_1_ and D_2_).

### 2.4. S1P Receptors Mediate the Effects of Exogenous and Endogenous S1P

**Figure 5:**
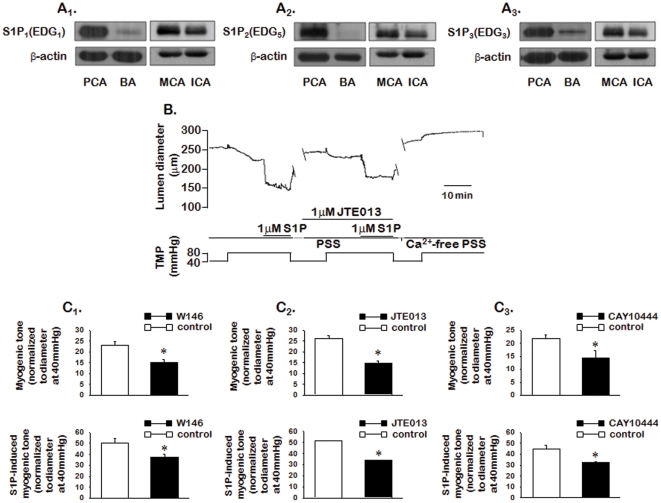
Expression of S1P receptors and effects of S1P receptor blockers on pressure- and exogenous S1P-induced myogenic tone. A: Western blot analysis of S1P receptor expression in rabbit vessels. Representative western blots of S1P receptors and actin in isolated rabbit posterior cerebral arteries (PCAs), basilar arteries (BAs), middle cerebral arteries (MCAs), and internal carotid arteries (ICA). B: Representative traces demonstrate changes in lumen diameter during the elevation of transmural pressure (from 40 to 80 mmHg) and application of S1P in the absence or presence of JTE013. C: Summarized data for changes in myogenic tone during the elevation of transmural pressure and application of S1P in the absence or presence of W146 (C_1_; n = 6), JTE013 (C_2_; n = 6), and CAY10444 (C_3_; n = 5). Data are expressed as means ± SEMs (n = 5–6). TMP: transmural pressure.

Three S1P receptors (S1P_1–3_; formerly known as Edg_1_, Edg_5_, and Edg_3_) are expressed in rabbit PCA (Figure 5A). These S1P receptors are also expressed in BAs, MCAs, and ICAs. To determine which S1P receptors are involved in endogenous and exogenous S1P-induced changes in myogenic tone, we evaluated myogenic tone in isolated rabbit PCAs in the presence and absence of specific S1P receptor blockers. As shown in Figures 5B and C, pretreatment with 1 µmol/L W146 (S1P_1_ blocker; Figure 5C_1_; n = 6), 1 µmol/L JTE013 (S1P_2_ blocker; Figure 5C_2_; n = 6), and 1 µmol/L CAY10444 (S1P_3_ blocker; Figure 5C_3_; n = 5) significantly (*P*<0.05) inhibited not only pressure-induced myogenic tone, but also S1P-induced myogenic tone. The extent of inhibition was similar among the S1P receptor blockers.

### 2.5. 20 kDa Myosin Light Chain ( MLC_20_) Phosphorylation and Myogenic Tone

**Figure 6:**
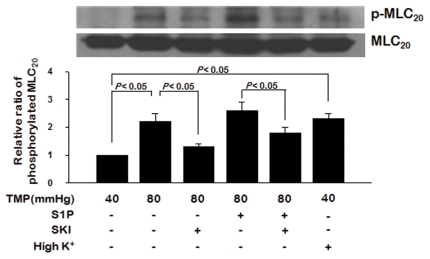
Changes in 20-KDa myosin light chain (MLC_20_) phosphorylation with the elevation of transmural pressure, and the effects of S1P or SKI application. Results are representative of immunoblots from five independent preparations. Results are expressed as means ± SEMs (n = 5). TMP: transmural pressure.

To determine the possible downstream effectors of myogenic tone, we measured MLC_20_ phosphorylation in steady-state myogenic tone. As shown in Figure 6, MLC_20_ phosphorylation increased significantly (*P*<0.05, n = 5) when the transmural pressure was raised from 40 to 80 mmHg. The level of MLC_20_ phosphorylation induced by pressure was similar to the high K^+^-induced MLC_20_ phosphorylation level. However, the pressure-induced increase in MLC_20_ phosphorylation was significantly inhibited (*P*<0.05, n = 5) by pre-treatment of the arteries with SKI. The pressure-induced increase in MLC_20_ phosphorylation was further increased by application of exogenous S1P when the pressure-induced myogenic tone had stabilized. SKI also significantly (*P*<0.05, n = 5) reduced the exogenous S1P-induced increase in MLC_20_ phosphorylation. However, the pressure-induced increase in MLC_20_ phosphorylation in arteries treated with S1P and SKI was higher than that in arteries treated with SKI alone.

## Discussion

A previous report indicated that the SK1, S1P-generating enzyme, can function as a mechanotransducer [12]–[14]. However, whether the SK1/S1P pathway was involved in myogenic vasoconstriction under normal physiological conditions was not determined. Furthermore, mechanisms underlying SK1/S1P pathway-induced myogenic tone were not fully elucidated.

The results of the present study suggest that the SK1/S1P pathway plays an important role in myogenic tone. The major findings of this study are as follows: 1) SK1 is expressed and activated in response to transmural pressure elevation in rabbit PCAs. Translocation of SK1 is dependent on Ca^2+^ influx via activation of voltage-sensitive Ca^2+^ channels following depolarization by activation of mechanosensitice ion channels; 2) pressure-induced myogenic tone is inhibited in rabbit PCAs treated with sphingosine kinase inhibitor (SKI), but augmented by treatment with NaF, an inhibitor of SPP1, NaF; 3) exogenous S1P further augments the pressure-induced myogenic response; 4) pressure induces an increase in Ca^2+^ concentration with the development of myogenic tone, and the increased Ca^2+^ concentration and myogenic tone is inhibited by SKI. Exogenous S1P further increases the pressure-induced increase in Ca^2+^ concentration and myogenic tone, but SKI has no effect. 5) pressure- and exogenous S1P-induced myogenic tone are inhibited by pre-treatment with fasudil, Rho kinase inhibitor, and NADPH oxidase inhibitors such as DPI and apocynin; 6) pressure- and exogenous S1P-induced myogenic tone are inhibited by pre-treatment with S1P receptor blockers W146 (S1P_1_ receptor blocker), JTE013 (S1P_2_ receptor blocker), and CAY10444 (S1P_3_ receptor blocker); and 7) MLC_20_ phosphorylation increases when transmural pressure is raised from 40 to 80 mmHg, while exogenous S1P further increases phosphorylation. The pressure-induced increase in MLC_20_ phosphorylation is inhibited by pre-treatment of arteries with SKI. Taken together, these results suggest that the SK1/S1P pathway may play an important role in pressure-induced myogenic responses in rabbit PCAs under normal physiological conditions.

To determine whether endogenous S1P generated in response to pressure was involved in myogenic vasoconstriction under normal physiological conditions, we first determined whether SK1 was expressed and activated by pressure in isolated rabbit PCA using western blotting and immunostaining. We detected SK1 proteins in isolated vessels including PCAs, BAs, MCAs, and ICAs and found that SK1 was translocated from the cytosol to the plasma membrane as a result of an increase in transmural pressure from 40 to 80 mmHg. SK1 translocation from the cytosol to the plasma membrane is an accepted feature of SK1 activation [12], [22]. To determine the mechanism involved in the translocation of SK1 by pressure elevation, we determined whether SK1 was translocated under several conditions. Translocation of SK1 by pressure elevation was blocked in the absence of external Ca^2+^ and in the presence of mechanosensitive ion channel blockers, amiloride (epithelial Na^+^ channel blocker) [23], 9-phenanthrol (selective TRPM4 blocker) [24], and SKF 96365 (nonselective blocker of TRPC channels) [25]. Translocation of SK1 by pressure elevation was also blocked in the presence of nifedipine, voltage-sensitive Ca^2+^ channel blocker. Although the blockers used in this study have many non-specific effects, the concentration used in the present study was reported to have no non-specific effect. Therefore, our results suggest that SK1 activity increases after membrane depolarization and Ca^2+^ influx via activation of voltage-sensitive Ca^2+^ channels, and are consistent with the previous findings that depolarization induces rapid and transient formation of intracellular S1P [26].

To determine whether endogenous S1P generated by pressure elevation plays a role in myogenic response, we evaluated the effect of SKI, an inhibitor of SK1, and NaF, an inhibitor of SPP1, on the pressure-induced myogenic response in rabbit PCA. Pressure-induced myogenic tone was inhibited in rabbit PCAs treated with SKI, but augmented by treatment with NaF. We also investigated the effect of exogenous S1P on myogenic tone to confirm the role of S1P in the myogenic response. Exogenous S1P further augmented the pressure-induced myogenic response. We also determined the effect of pre-treatment of exogenous S1P on the SKI-induced inhibitory effect of myogenic tone. Pre-treatment of S1P decreased blood vessel diameter and abolished SKI-induced inhibitory effect of myogenic tone. Taken together, our results suggest that the elevation of transmural pressure activates SK1 and then increases endogenous S1P. Furthermore, our results suggest that endogenous S1P generated by pressure elevation is involved in the development of myogenic tone in PCAs and are consistent with the previous findings that SK1/S1P plays a specific role as a modulator of cerebral blood flow [27].

It is well known that endothelial cells modulate vascular tone by releasing nitric oxide. Interestingly, it has previously reported that SK1 increases nitric oxide production by activation of endothelial nitric oxide synthase [28]. In the present study, to determine the role of endothelium on the SK1/S1P-induced myogenic tone, we measured endogenous and exogenous S1P-induced myogenic tone in PCAs with and without endothelial cells. We found that pressure- and S1P-induced myogenic tone were not different between endothelium denuded and intact arteries. We also found that the SKI-induced inhibitory effect of myogenic tone was similar in endothelium denuded and intact arteries.

It is generally accepted that myogenic vasoconstriction is mediated by a combination of elevation of cytosolic Ca^2+^ concentration ([Ca^2+^]_i_) [29], [30] and Ca^2+^ sensitization [20]. We have previously reported that S1P-induced vasoconstrictions are mediated by a combination of Ca^2+^ mobilization from the sarcoplasmic reticulum and Ca^2+^ influx through L-type Ca^2+^ channels in addition to a Ca^2+^ sensitization mechanism [19]. In the present study, to evaluate whether increases in [Ca^2+^]_i_ and/or the Ca^2+^ sensitization pathway contribute to the endogenous S1P-induced myogenic tone, we first measured the effect of SKI on changes in [Ca^2+^]_i_ and myogenic tone when the transmural pressure was elevated. We found that pressure induced an increase in [Ca^2+^]_i_ with subsequent development of myogenic tone, and the increased [Ca^2+^]_i_ and myogenic tone were inhibited by SKI. We also showed that exogenous S1P further increased the pressure-induced increased [Ca^2+^]_i_ and myogenic tone, but SKI had no effect. These results suggest that the increase in [Ca^2+^]_i_ involved in myogenic vasoconstriction is mediated by endogenous S1P generated in response to pressure elevation. However, we did not determine the source of the [Ca^2+^]_i_ increase induced by endogenous S1P.

We investigated the role of the Ca^2+^ sensitization mechanism in endogenous S1P-induced myogenic tone, using fasudil, DPI, and apocynin to assess the influence of Rho A/Rho kinase and NADPH oxidase-dependent generation of ROS. Fasudil inhibited pressure- and exogenous S1P-induced myogenic tone. DPI and apocynin also inhibited pressure- and exogenous S1P-induced myogenic tone. These results suggest that the RhoA/Rho-kinase and ROS-mediated Ca^2+^ sensitization mechanisms play an important role in S1P-induced myogenic tone. Our results are consistent with the previous findings that S1P-mediated activation of the RhoA/Rho kinase pathway is an integral part of myogenic tone [13] and NADPH oxidase-derived ROS production is increased in response to the elevation of transmural pressure [31].

Although a link between SK1/S1P and Rho A/Rho kinase or NADPH oxidase has been identified, we did not identify the specific signaling mechanisms that allow for their connection. S1P is a pleiotropic mediator and can act as both an intracellular second messenger and an extracellular ligand. The exact signaling targets of intracellular S1P remain unidentified but extracellular S1P signals are transduced by five distinct G-protein coupled receptors (S1P1–5), which can activate small GTPases (e.g., RhoA and Rac), leading to NADPH oxidase activation [12]. In the present study, we investigated the role of S1P receptors in endogenous and exogenous S1P-induced myogenic tone. Although five S1P receptors (S1P_1–5_) have been identified [32], three specific receptors (S1P_1–3_) are reportedly expressed at the mRNA level in vascular smooth muscle [33]. We verified that S1P_1–3_ are expressed at the protein level using western blot. To determine the role of S1P receptors in endogenous and exogenous S1P-induced myogenic tone, we tested the effects of S1P receptor specific blockers such as W146 (S1P_1_ receptor blocker), JTE013 (S1P_2_ receptor blocker), and CAY10444 (S1P_3_ receptor blocker) on endogenous and exogenous S1P-induced myogenic tone. All three type-specific receptor blockers significantly inhibited both pressure- (endogenous S1P) and exogenous S1P-induced myogenic tone. These results suggest that endogenous S1P generated in response to the elevation of pressure may act not only as an intracellular second messenger, but also as an extracellular ligand after being transported across the plasma membrane. Intracellularly generated S1P is unable to move through hydrophobic mammalian cell plasma membranes since it possesses a polar head group. Although the mechanism of S1P release from cells is not completely understood, the involvement of the ATP-binding cassette (ABC) family of transporters, especially ABCC7 (CFTR), has been suggested [14].

Smooth muscle contraction is activated primarily by phosphorylation at Ser^19^ of the 20 kDa regulatory light chain of myosin II. Therefore, to directly prove that SK1/S1P plays an important role in pressure-induced myogenic constriction under normal physiological conditions, it is very important to determine the changes in MLC_20_ phosphorylation during the development of myogenic responses and the effect of SK1/S1P blockade on MLC_20_ phosphorylation. In the present study, we observed an increase in MLC_20_ phosphorylation in rabbit PCAs when transmural pressure or exogenous S1P was applied. The pressure-induced increase in MLC_20_ phosphorylation was inhibited by pretreatment with SKI. Thus, the data suggest that enhanced MLC_20_ phosphorylation in response to endogenous S1P generated by transmural pressure elevation plays a central role in the modulation of myogenic tone.

Based on the results of the present study, it is clear that endogenous S1P-induced myogenic tone can be regulated by Ca^2+^-dependent and/or Ca^2+^-independent (Ca^2+^-sensitization) mechanisms. Increased transmural pressure activates mechanosensitive ion channels that putatively lead to Ca^2+^ influx via voltage-sensitive Ca^2+^ channels and activation of SK1. SK1 converts sphingosine to S1P. Endogenous and/or exogenous S1P then increase the intracellular Ca^2+^ concentration and myosin light chain phosphorylation via activation of myosin light chain kinase. On the other hand, extracellular S1P acts as a receptor ligand and activates several signaling pathways, including RhoA/Rho kinase and Rac. RhoA/Rho kinase increases apparent Ca^2+^ sensitivity by inhibiting myosin light chain phosphatase (MLCP). The activation of Rac is associated with increased formation of O2 – via NADPH oxidase. This pathway also modulates the apparent Ca^2+^ sensitivity by inhibiting MLCP.

In summary, our results suggest that the SK1/S1P pathway may play an important role in pressure-induced myogenic responses in rabbit PCAs under normal physiological conditions. S1P generated through SK1 activation by pressure increases myogenic tone. The underlying mechanisms for endogenous S1P-induced myogenic tone are an increase in [Ca^2+^]_i_ and the Ca^2+^ sensitization mechanism via Rho A/Rho kinase and NADPH oxidase/ROS. Because endogenous S1P and ROS production is elevated under pathophysiological conditions such as hypertension, atherosclerosis, and vasospasm, the SK1/S1P pathway likely plays an important role in myogenic tone under pathophysiological conditions.
